# Catalytic Oxidation of NO over MnO_x_–CeO_2_ and MnO_x_–TiO_2_ Catalysts

**DOI:** 10.3390/molecules21111491

**Published:** 2016-11-14

**Authors:** Xiaolan Zeng, Xiaoyue Huo, Tianle Zhu, Xiaowei Hong, Ye Sun

**Affiliations:** School of Space and Environment, Beihang University, Beijing 100191, China; xlzeng@buaa.edu.cn (X.Z.); xyhuo1123@163.com (X.H.); hongxiao86@126.com (X.H.); suny@buaa.edu.cn (Y.S.)

**Keywords:** MnO_x_–CeO_2_, MnO_x_–TiO_2_, catalytic oxidation, NO, SO_2_

## Abstract

A series of MnO_x_–CeO_2_ and MnO_x_–TiO_2_ catalysts were prepared by a homogeneous precipitation method and their catalytic activities for the NO oxidation in the absence or presence of SO_2_ were evaluated. Results show that the optimal molar ratio of Mn/Ce and Mn/Ti are 0.7 and 0.5, respectively. The MnO_x_–CeO_2_ catalyst exhibits higher catalytic activity and better resistance to SO_2_ poisoning than the MnO_x_–TiO_2_ catalyst. On the basis of Brunauer–Emmett–Teller (BET), X-ray diffraction (XRD), and scanning transmission electron microscope with mapping (STEM-mapping) analyses, it is seen that the MnO_x_–CeO_2_ catalyst possesses higher BET surface area and better dispersion of MnO_x_ over the catalyst than MnO_x_–TiO_2_ catalyst. X-ray photoelectron spectroscopy (XPS) measurements reveal that MnO_x_–CeO_2_ catalyst provides the abundance of Mn^3+^ and more surface adsorbed oxygen, and SO_2_ might be preferentially adsorbed to the surface of CeO_2_ to form sulfate species, which provides a protection of MnO_x_ active sites from being poisoned. In contrast, MnO_x_ active sites over the MnO_x_–TiO_2_ catalyst are easily and quickly sulfated, leading to rapid deactivation of the catalyst for NO oxidation. Furthermore, temperature programmed desorption with NO and O_2_ (NO + O_2_-TPD) and in situ diffuse reflectance infrared transform spectroscopy (in situ DRIFTS) characterizations results show that the MnO_x_–CeO_2_ catalyst displays much stronger ability to adsorb NO_x_ than the MnO_x_–TiO_2_ catalyst, especially after SO_2_ poisoning.

## 1. Introduction

Nitrogen oxides (NO_x_) emitted from stationary and mobile sources are some of the main air pollutants, which cause a variety of serious environmental problems, such as photochemical smog, acid rain, and greenhouse effect [1]. Moreover, NO_x_ are the primary precursors of haze occurring in China. Therefore, NO_x_ removal has become the focus of recent environmental protection. The most effective and mature technology is the selective catalytic reduction using ammonia as a reducing agent (NH_3_-SCR). However, there still exist some problems, such as high reaction temperature, sophisticated system design, and high operation cost. Additionally, it is possible to cause secondary pollution due to the leakage of ammonia [2].

In order to solve the problems of NH_3_-SCR, much attention has been paid to the simultaneous removal of SO_2_ and NO_x_ by chemical absorption. For the absorption operation, the oxidation of NO with low water-solubility to NO_2_ is a crucial process because NO accounts for about 95% of NO_x_. In general, the oxidation of NO to NO_2_ can be realized through gas phase oxidation and liquid phase oxidation. The presence of SO_2_ is disadvantageous to NO oxidation in the liquid phase because of the high solubility and oxidizability of SO_2_, whereas the oxidation rate of SO_2_ is much lower than that of NO in the gas phase [3]. The gas phase oxidation is divided into homogeneous gas phase oxidation and heterogeneous gas-solid catalytic oxidation. Nowadays, catalytic oxidation of NO is potentially an ideal technology due to its simple operation and low cost, and considerable interest has been put into the investigation of developing catalysts for oxidizing NO into NO_2_.

The catalysts for NO oxidation mainly include noble metal catalysts, transition metal catalysts, and molecular sieve catalysts. Noble metal catalysts exhibit high catalytic activity at low temperature, but are limited in industrial applications because of their high cost and poisoning problems [4,5,6,7,8,9]. Molecular sieve catalysts show certain catalytic activity but they are hydrothermally unstable and susceptible to structure collapse [10]. Transition metal oxides are cheap and also have good catalytic activity and, thus, can be appropriate catalysts for the catalytic oxidation of NO. Among the variety of transition metal catalysts, Co-based and Mn-based catalysts display the best catalytic activity for NO oxidation [11]. However, the applications of Co-based catalysts are retarded due to the toxicity of cobalt although they attract much attention [12,13,14,15,16,17,18,19]. Mn-based catalysts are considered as the promising candidates for NO oxidation to NO_2_. Many Mn-based catalysts (e.g., MnO_x_/TiO_2_ [20,21,22], Ce–Mn/TiO_2_ [23], FeMnO_x_/TiO_2_ [24,25]) have been studied. The results show that MnO_x_ supported on TiO_2_ (P25) prepared by deposition-precipitation (DP) method and chemical vapor condensation method exhibits high catalytic activity. Additionally, NO oxidation efficiency can be enhanced by modifying MnO_x_/TiO_2_ with Ce and Fe. Most recently, many Mn-based catalysts (e.g., Mn–Ce–Ti [26], MnO_x_/CeO_2_–ZrO_2_ [27], MnO_2_/TiO_2_–Pal [28], Co–Mn/TiO_2_ [29], Fe_2_O_3_@MnO_x_@CNTs [30], and MnO_2_@NiCo_2_O_4_ [31]) have also been studied on the selective catalytic reduction of NO_x_, and they exhibit good catalytic activities. On the other hand, CeO_2_, as a carrier or promoter, also has been studied extensively because of its redox properties and exceptional ability to store and release oxygen. Meanwhile, studies also show that CeO_2_ possesses excellent ability to resist SO_2_ poisoning [23,32].

In this study, we compared the catalytic activity and resistance to SO_2_ poisoning of MnO_x_–CeO_2_ and MnO_x_–TiO_2_ catalysts. The fresh and SO_2_ poisoned catalysts were characterized by XRD, BET, STEM-mapping, XPS, NO + O_2_-TPD and in situ DRIFTS to clarify the structure-effect relationship.

## 2. Results and Discussion

### 2.1. Catalytic Activity Tests

**Figure 1:**
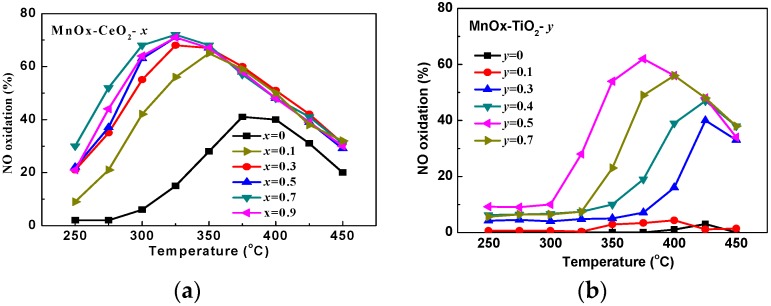
NO oxidation over MnO_x_–CeO_2_-*x* catalysts (**a**) and MnO_x_–TiO_2_-*y* catalysts (**b**). Reaction conditions: 400 ppm NO, 10% O_2_, 1% H_2_O, 100 ppm SO_2_, balanced with N_2_; GHSV = 40,000 h^−1^.

The NO oxidation efficiencies over the MnO_x_–CeO_2_-*x* and MnO_x_–TiO_2_-*y* catalysts are shown in Figure 1a,b, respectively. It can be seen that TiO_2_ shows negligible catalytic activity during the reaction temperature range, while CeO_2_ has certain catalytic activity for NO oxidation. Nonetheless, the catalytic activity of CeO_2_ is lower and the activity temperature is higher, compared with those of MnO_x_–CeO_2_-*x* catalysts. Therefore, MnO_x_ was the main active component for the catalytic oxidation of NO. In the presence of SO_2_, the optimal molar ratio of Mn/Ce and Mn/Ti was 0.7 and 0.5, respectively. Meanwhile, the maximum NO oxidation efficiency of 72% over MnO_x_–CeO_2_-0.7 catalyst is obtained at 325 °C, while that of 62% over MnO_x_–TiO_2_-0.5 catalyst is obtained at 375 °C Therefore, the MnO_x_–CeO_2_-0.7 catalyst has better catalytic activity than the MnO_x_–TiO_2_-0.5 catalyst.

**Figure 2:**
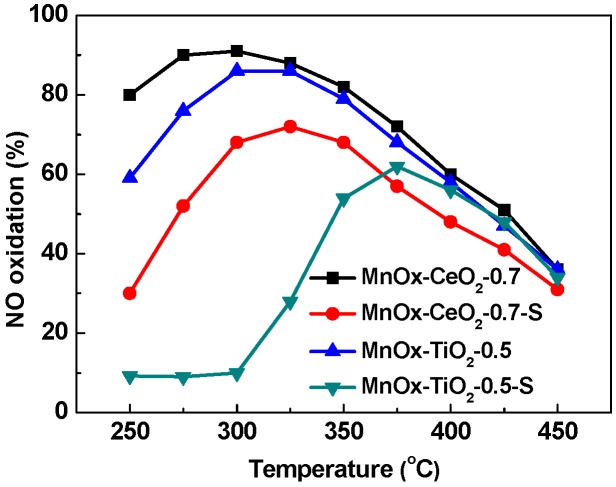
The effect of SO_2_ on NO oxidation over MnO_x_–CeO_2_-0.7 and MnO_x_–TiO_2_-0.5 catalysts. Reaction conditions: 400 ppm NO, 10% O_2_, 1% H_2_O, 100 ppm SO_2_ (when used), balanced with N_2_; GHSV = 40,000 h^−1^.

Actually, the catalytic activities of MnO_x_–CeO_2_-0.7 and MnO_x_–TiO_2_-0.5 catalysts were also investigated in the absence of SO_2_, and the results show that the maximum NO oxidation efficiency of MnO_x_–CeO_2_-0.7 and MnO_x_–TiO_2_-0.5 catalysts are 91% and 86% at 300 °C, as shown in Figure 2. Clearly, the presence of SO_2_ results in a decrease of NO oxidation efficiency and an increase of the active temperature, especially for the MnO_x_–TiO_2_-0.5 catalyst. The MnO_x_–CeO_2_-0.7 catalyst displays better resistance to SO_2_ poisoning than the MnO_x_–TiO_2_-0.5 catalyst.

**Figure 3:**
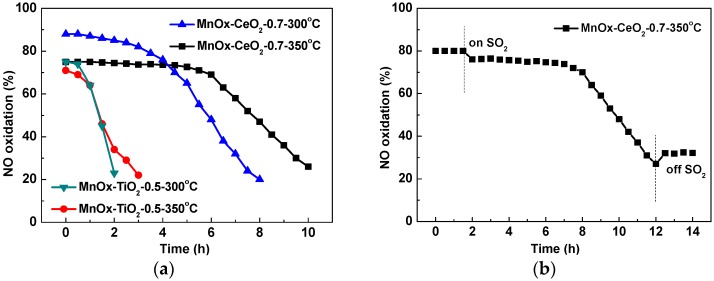
The stability test for NO oxidation over MnO_x_–CeO_2_-0.7 and MnO_x_–TiO_2_-0.5 catalysts (**a**); The effect of on-off of SO_2_ over MnO_x_–CeO_2_-0.7 catalyst (**b**). Reaction conditions: 400 ppm NO, 10% O_2_, 1% H_2_O, 100 ppm SO_2_ (when used), balanced with N_2_; GHSV = 40,000 h^−1^.

The stability tests for NO oxidation over MnO_x_–CeO_2_-0.7 and MnO_x_–TiO_2_-0.5 catalysts were carried out under different temperatures. As shown in Figure 3a, the NO oxidation efficiency of the MnO_x_–TiO_2_-0.5 catalyst decreases much more rapidly than that of the MnO_x_–CeO_2_-0.7 catalyst. The catalytic activity of the MnO_x_–CeO_2_-0.7 catalyst gradually decreases at 300 °C, and maintains almost unchanged within 5 h at 350 °C while it decreases after 5 h. The stability tests without SO_2_ over two catalysts were also carried out at 300 °C, and no activity decrease is observed in 20 h (the results are not shown here), which convinces us that the deactivation in Figure 3 is caused by the presence of SO_2_. The on-off effect of SO_2_ for NO oxidation over MnO_x_–CeO_2_-0.7 catalyst was investigated. As shown in Figure 3b, when 100 ppm SO_2_ are added to the reactants, the NO oxidation efficiency decreases from the initial 80% to 27% after 10 h. After excluding SO_2_ from the flue gas, the NO oxidation efficiency only recovers to 32%, which indicates that the poisoning effect of SO_2_ is irreversible.

### 2.2. XRD and BET Characterizations

**Figure 4:**
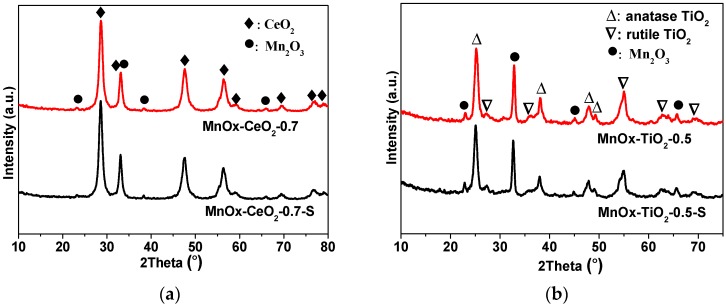
XRD patterns of MnO_x_–CeO_2_-0.7 and MnO_x_–CeO_2_-0.7-S catalysts (**a**) and MnO_x_–TiO_2_-0.5 and MnO_x_–TiO_2_-0.5-S catalysts (**b**).

Figure 4 presents the XRD patterns of fresh and SO_2_ poisoned catalysts. For MnO_x_–CeO_2_-0.7 catalyst and SO_2_ poisoned MnO_x_–CeO_2_-0.7 catalyst (donated as MnO_x_–CeO_2_-0.7-S), crystalline phases of CeO_2_ can be clearly observed, and very weak signals of Mn_2_O_3_ are also detected, which indicates that Mn_2_O_3_ exists in a poor crystal structure. For MnO_x_–TiO_2_-0.5 catalyst and SO_2_ poisoned MnO_x_–TiO_2_-0.5 catalyst (donated as MnO_x_–TiO_2_-0.5-S), the stronger diffraction peaks of Mn_2_O_3_ are observed besides crystalline phases of rutile and anatase TiO_2_, which suggests that Mn_2_O_3_ exists in crystal structure. It is well know that the low crystallinity of MnO_x_ is favorable for catalytic reaction [20]. Therefore, the higher activity of MnO_x_–CeO_2_-0.7 catalyst may be partly due to the well dispersion of MnO_x_. For all of the samples, the diffraction peaks almost do not change due to SO_2_ poisoning.

The BET surface areas of the catalysts are summarized in Table 1. It can be seen that the specific surface areas of fresh MnO_x_–CeO_2_-0.7 and MnO_x_–TiO_2_-0.5 catalysts are 96.30 and 60.21 m^2^·g^−1^. Compared to catalytic performance, it is consistent with that of BET surface. Furthermore, it is worth noting that the BET specific surface areas of SO_2_ poisoned catalysts decrease to 67.92 (MnO_x_–CeO_2_-0.7) m^2^·g^−1^ and 39.71 (MnO_x_–TiO_2_-0.5) m^2^·g^−1^, which may be caused by the formation of sulfate species.

### 2.3. STEM-Mapping Analysis

**Figure 5:**
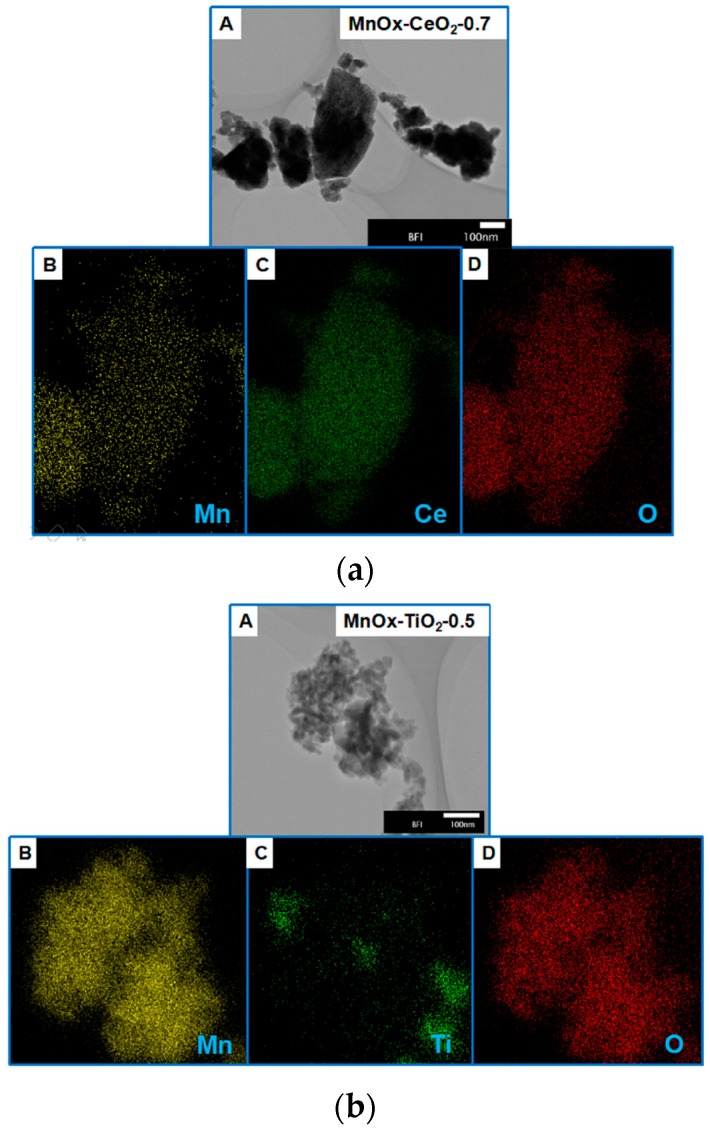
STEM images and their mapping analyses of MnO_x_–CeO_2_-0.7 catalyst (**a**) and MnO_x_–TiO_2_-0.5 catalyst (**b**). (**A**) STEM images of MnO_x_–CeO_2_-0.7 or MnO_x_–TiO_2_-0.5 catalysts; (**B**) Mn; (**C**) Ce or Ti; and (**D**) O.

Figure 5 presents STEM images and their mapping analysis of fresh MnO_x_–CeO_2_-0.7 and MnO_x_–TiO_2_-0.5 catalysts. For the MnO_x_–CeO_2_-0.7 catalyst, Mn, Ce, O evenly disperses on the scanning area, which indicates excellent distribution of MnO_x_ and CeO_2_. For the MnO_x_–TiO_2_-0.5 catalyst, however, many of the Mn and O atoms appear on the scanning area, while few Ti atoms are seen. Therefore, we deduce that TiO_2_ cannot disperse MnO_x_ well, which can lead to low catalytic activity of the MnO_x_–TiO_2_-0.5 catalyst.

### 2.4. XPS Analysis

**Figure 6:**
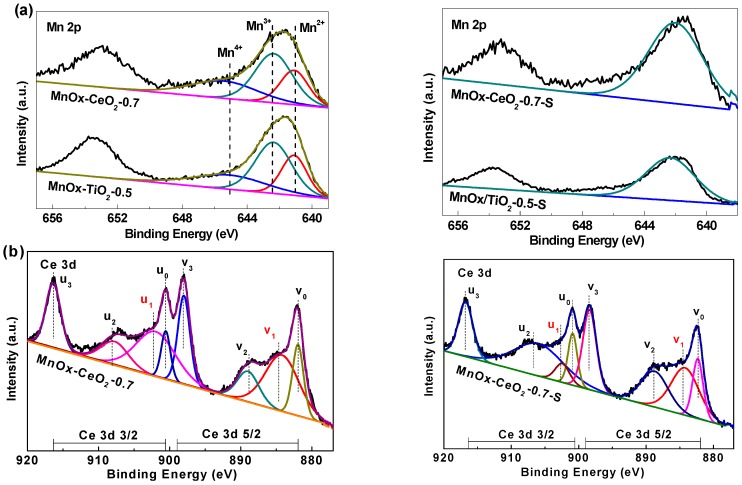

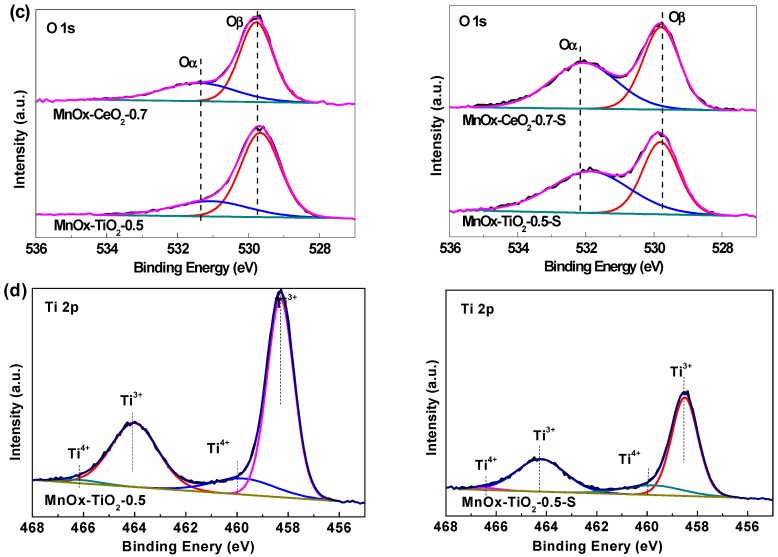
XPS spectra of the catalysts. (**a**) Mn 2p; (**b**) Ce 3d; (**c**) O 1s; and (**d**) Ti 2p.

XPS analysis was performed to identify the surface component and chemical states of fresh and SO_2_ poisoned catalysts. Surface atomic concentration and ratio are summarized in Table 2, and XPS spectra of Mn 2p, O 1s, Ce 3d, and Ti 2p of all catalysts are displayed in Figure 6. Through the deconvolution of the spectra, two main peaks due to Mn 2p_1/2_ and Mn 2p_3/2_ are observed. The Mn 2p_3/2_ profiles are fitted with the Mn^2+^, Mn^3+^, and Mn^4+^, characterized by the binding energy at about 641.1 eV, 642.5 eV, and 645.1 eV [33], respectively. Previous studies [20,22,34] have shown that Mn_2_O_3_ has a higher catalytic activity than MnO_2_ for NO oxidation. Cimino et al. [35] attributed the higher activity of Mn^3+^ than Mn^4+^ for CO catalytic oxidation to the weaker Mn^3+^–O bond. Similarly, it can be deduced that the weaker Mn^3+^–O bonds will also favor the catalytic oxidation of NO since the Mn^3+^–O bond is easily broken, thus, promoting the generation and release of the NO_2_ oxidation product. As shown in Table 2, all catalysts contain high concentration of Mn^3+^. Corresponding to the high catalytic activity of catalyst, it, combining with XRD analysis results, can be also speculated that Mn^3+^ has higher catalytic activity than Mn^2+^ and Mn^4+^ for NO oxidation. The Ce 3D XPS spectra can be separated into eight peaks: *u*_0_ (900.6 eV), *u*_1_ (902.4eV), *u*_2_ (907.9 eV), *u*_3_ (916.6 eV), *v*_0_ (881.9 eV), *v*_1_ (884.4 eV), *v*_2_ (889.1 eV), and *v*_3_ (898.1 eV) [36]. The bands labeled as *u*_1_ and *v*_1_ are attributed to Ce^3+^ species, and the other six peaks are assigned to Ce^4+^ species. The ratio of Ce^3+^/(Ce^3+^ + Ce^4+^) can be estimated by the formula [37]:

$${\mathrm{Ce}}^{3+}(\%)=\frac{S_{u_{1}}+S_{v_{1}}}{\sum_{i=0}^{3}\left(S_{u_{i}}+S_{v_{i}}\right)}\;\times \;100\%$$

It is well known that Ce^3+^ species can make charge imbalance and create oxygen vacancies via the shift from Ce^3+^ to Ce^4+^, which leads to the increase of surface adsorbed oxygen (Ce^3+^ → Ce^4+^ + e^−^, O_2_ + e^−^ → O_2_^−^) [38]. For the catalytic oxidation of NO, surface adsorbed oxygen plays a significant role because of its mobility and redox performance [39]. As listed in Table 2, the Ce^3+^ concentration can reach about 41.6%. Figure 6c displays the O 1s XPS spectra of all samples, two distinct bands are obtained. The one peak O_β_ in the range of 528–530 eV belongs to lattice oxygen and the other peak O_α_ with binding energy of 530–532 eV corresponds to weakly surface adsorbed oxygen [18]. From Table 2, it can be seen that the O_α_ concentration over MnO_x_–CeO_2_-0.7 catalyst is higher than that over MnO_x_–TiO_2_-0.5 catalyst, which is attributed to the presence of Ce^3+^ species.

On the other hand, the Mn concentration of the MnO_x_–CeO_2_-0.7-S catalyst is almost the same to that of the fresh MnO_x_–CeO_2_-0.7 catalyst, while the Mn concentration of MnO_x_–TiO_2_-0.5 catalyst and the Ce concentration of MnO_x_–CeO_2_-0.7 catalyst decrease from 13.6% to 9.4% and from 26.4% to 19.7%, respectively, because of SO_2_ poisoning, which is attributed that the MnO_x_ over MnO_x_–TiO_2_-0.5-S catalyst and CeO_2_ over MnO_x_–CeO_2_-0.7-S catalyst are partly covered with sulfate species [36]. Meanwhile, the ratio of Ce^3+^/(Ce^3+^ + Ce^4+^) of the MnO_x_–CeO_2_-0.7 catalyst also decreases from 41.6% to 25.2%, which indicates that cerium(IV) sulfate may be formed on the catalyst surface [40]. Therefore, we can deduce that SO_2_ might be preferentially adsorbed to the surface of CeO_2_ to form sulfate species, lessening the sulfation of MnO_x_ active sites. It was also reported by Jin and co-workers [32] that the presence of CeO_2_ might partially prevent MnO_x_ active sites from being sulfated. Waqif [41] investigated the adsorption of SO_2_ on CeO_2_–Al_2_O_3_, and concluded that ceria was a basic material for SO_2_ adsorption. Figure 4d shows the Ti 2p XPS spectra, four peaks are formed, referred to as Ti^3+^ at 458.3 eV, 464.1 eV, and Ti^4+^ at 459.8 eV, 466.1 eV, respectively [23]. Though the Ti^3+^ concentration is pretty high, it still cannot improve the resistance to SO_2_ poisoning.

### 2.5. NO + O_2_-TPD and In Situ DRIFTS Analyses

**Figure 7:**
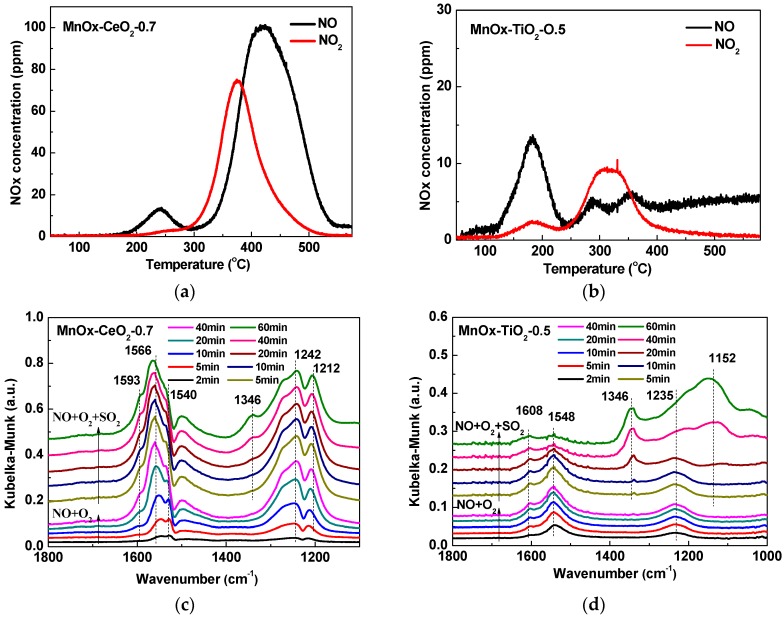
NO + O_2_-TPD profiles of the MnO_x_–CeO_2_-0.7 catalyst (**a**) and the MnO_x_–TiO_2_-0.5 catalyst (**b**); in situ DRIFTS spectra of MnO_x_–CeO_2_-0.7 catalyst (**c**); and MnO_x_–TiO_2_-0.5 catalyst (**d**) at 350 °C.

The adsorption behavior of the catalyst is considered a crucial step in a catalytic oxidation reaction. Therefore, NO + O_2_-TPD experiments were conducted to explore the NO_x_ adsorption ability over MnO_x_–CeO_2_-0.7 and MnO_x_–TiO_2_-0.5 catalysts. As shown in Figure 7a,b, the NO and NO_2_ curves over MnO_x_–CeO_2_-0.7 and MnO_x_–TiO_2_-0.5 catalysts are observed. For the MnO_x_–CeO_2_-0.7 catalyst, the desorption peak at about 240 °C is assigned to nitrosyl species [42], the desorption peak in the temperature range of 350–450 °C can be ascribed to the decomposition of strong adsorption species such as nitrate on catalyst surface [43]. For the MnO_x_–TiO_2_-0.5 catalyst, three major desorption peaks at 80, 180, and 320 °C are observed, which may be attributed to desorption of molecularly-adsorbed NO and NO_2_, nitrosyl species and desorption of nitrate species, respectively [42,44,45]. It is obvious that the total amount of NO_x_ desorbed from MnO_x_–CeO_2_-0.7 catalyst is remarkably larger than that of the MnO_x_–TiO_2_-0.5 catalyst, indicating stronger adsorption and oxidation abilities on the surface of the MnO_x_–CeO_2_-0.7 catalyst.

In order to understand the NO_x_ adsorption behaviors and SO_2_ poisoning process, in situ DRIFTS measurements over MnO_x_–CeO_2_-0.7 and MnO_x_–TiO_2_-0.5 catalysts were carried out at 350 °C. Figure 7c,d shows the NO-O_2_ co-adsorption accompanied by SO_2_ adsorption. After introducing NO + O_2_, for the MnO_x_–CeO_2_-0.7 catalyst, the bands at 1593, 1566, 1540, 1242, and 1212 cm^−1^ are detected. All of the bands’ intensities gradually increase with the adsorption time until reaching their highest intensities and remain stable after about 40 min. The bands at 1566, 1540, and 1212–1242 cm^−1^ are assigned to bidentate nitrate, monodentate nitrate, and bridge nitrate, respectively [46]. A very weak band at 1593 cm^−1^ is due to the adsorption of NO_2_ [47]. For the MnO_x_–TiO_2_-0.5 catalyst, the bands attributed to monodentrate nitrate (1235 cm^−1^), bidentrate nitrate (1548 cm^−1^), and bridge nitrate (1608 cm^−1^) are observed [44]. The change trend of these bands’ intensities is similar to those over the MnO_x_–CeO_2_-0.7 catalyst. However, it is obvious that all of the adsorption bands’ intensities of the MnO_x_–TiO_2_-0.5 catalyst are significantly lower than those of the MnO_x_–CeO_2_-0.7 catalyst, which is probably one of the reasons that the MnO_x_–CeO_2_-0.7 catalyst has better activity than the MnO_x_–TiO_2_-0.5 catalyst in the absence of SO_2_.

In the following, 100 ppm SO_2_ was added to the reaction system. It can be seen from Figure 7c that a new band at 1346 cm^−1^ appears over the MnO_x_–CeO_2_-0.7 catalyst and the intensity grows with time. Similarly, the new peaks at 1346 cm^−1^ and 1152 cm^−1^ are also observed over the MnO_x_–TiO_2_-0.5 catalyst and their intensities rise rapidly with the reaction time. The band at 1346 cm^−1^ is due to the ʋ (S=O) vibration of surface sulfate species, and the band at 1152 cm^−1^ can be ascribed to sulfate species [48]. Moreover, it can be noted that all the adsorption bands’ intensities almost remain unchanged within 10 min and the bands’ intensities of monodentrate and bidentrate nitrate slightly decrease for the MnO_x_–CeO_2_-0.7 catalyst. However, the bands’ intensities at 1608 cm^−1^ and 1548 cm^−1^ drop rapidly with time and the peak of monodentrate nitrate almost vanishes after 60 min for the MnO_x_–TiO_2_-0.5 catalyst. The results confirm that SO_2_ has little influence on NO_x_ adsorption over the MnO_x_–CeO_2_-0.7 catalyst, while there is strongly competitive adsorption between SO_2_ and NO_x_ over the MnO_x_–TiO_2_-0.5 catalyst in a certain reaction time. Tang et al. [49] reported the mechanism of catalytic oxidation of NO over Mn-based catalysts that NO firstly adsorbed on Mn sites to form nitrosyls, and then were oxidized to nitrates, which decomposed to the final product, NO_2_.

According to the DRIFTS results and the mechanism, we further deduce that SO_2_ preferentially combines with CeO_2_ to form sulfate species, and MnO_x_ active sites are exposed to the surface to adsorb NO_x_ over the MnO_x_–CeO_2_-0.7 catalyst. Whereas MnO_x_ active sites are sulfated so seriously that the MnO_x_–TiO_2_-0.5 catalyst has no ability to adsorb NO_x_, leading to low catalytic activity. The results are consistent with XPS analysis. Moreover, the formation of sulfate species is irreversible and sulfate species occupied the sites for NO oxidation permanently. Through the above analysis, it is sufficient to prove that the catalytic activity and resistance to SO_2_ poisoning of MnO_x_–CeO_2_ catalysts are better than MnO_x_–TiO_2_ catalysts.

## 3. Materials and Methods

### 3.1. Catalyst Preparation

A series of MnO_x_–CeO_2_-*x* and MnO_x_–TiO_2_-*y* catalysts, where *x* and *y* are the molar ratio of Mn/Ce and Mn/Ti, respectively, were prepared by homogeneous precipitation method. Take MnO_x_–CeO_2_-0.7 for example, 13.02 g Ce(NO_3_)_3_·6H_2_O and 7.15 g Mn(NO_3_)_2_ (50% solution) were firstly added to 100 mL deionized water and stirred for 2 h. Excessive urea aqueous solution was added into the mixed solution under stirring. Then, the mixed solution was stirred for 12 h at 90 °C. In order to make Mn precipitate completely, an appropriate amount of ammonia solution were added into the mixed solution until the pH value was 9.5. The precipitate was collected by filtration and washed with deionized water, followed by drying at 110 °C overnight and subsequently calcination at 500 °C for 4 h in the air atmosphere. MnO_x_–TiO_2_-*y* catalysts were prepared by similar process with MnO_x_–CeO_2_-*x* catalysts. The difference is that tetrabutyl titanate was firstly dissolved in ethanol. Finally, the catalysts were crushed and sieved to 40–60 mesh for activity test.

### 3.2. Catalytic Activity Measurement

The catalytic activity was evaluated in a quartz U-tube fixed-bed flow reactor (i.d. 13 mm) from 450–250 °C. The test data was recorded after the reaction for 40 min at each temperature. The reaction gas consisted of 400 ppm NO, 10% O_2_, 1% H_2_O, 100 ppm SO_2_ (when used), and balanced with N_2_. The total flow rate was fixed at 2 L/min, which is corresponded to a gas hourly space velocity (GHSV) of 40,000 h^−1^. The concentrations of NO, NO_2_, O_2_, and SO_2_ were analyzed by a flue gas analyzer (Testo 350, Testo AG, Schwarzwald, Germany). The NO oxidation efficiency was calculated by the following equation:

$$\mathrm{NO}\;\mathrm{oxidation}\;(\%)=\frac{{\left[\mathrm{NO}\right]}_{\mathrm{inlet}}-{\left[\mathrm{NO}\right]}_{\mathrm{outlet}}}{{\left[\mathrm{NO}\right]}_{\mathrm{inlet}}}\times 100\%$$

### 3.3. Catalyst Characterization

The XRD patterns were recorded by powder X-ray diffractometer (XRD-600) with Cu Kα radiation (λ = 1.54 Å). The samples were scanned at 2θ ranging from 10° to 80° with a scan speed of 6° min^−1^. BET Surface areas of the catalysts were determined by N_2_ adsorption-desorption isotherms at −196 °C using specific surface area and porosity analyzer (NOVA 2200, Quantachrome, Boynton Beach, FL, USA). The samples were degassed under vacuum at 300 °C for 4 h. The STEM-mapping analysis was performed using a transmission electron microscope (JEM-2100F, JEOL, Tokyo, Japan) to observe distribution of metal oxides. The surface chemical states of catalysts were tested by X-ray photoeletron spectra (PHI Quantro SXM^TM^, ULVAC-PHI, Kanagawa, Japan) using an Al Kα X-ray source (1486.7 eV) at 15 kV and 25 W with the binding energy calibrated by C 1s at 284.8 eV.

The NO + O_2_-TPD experiments were performed in a quartz reactor with a FTIR spectrometer (MultiGas^TM^ 2030 HS). Prior to the tests, the samples (200 mg) were pretreated in 10% O_2_/N_2_ (500 mL/min) at 500 °C 0.5 h followed by cooling down to 350 °C. The catalysts were exposed to 400 ppm NO, 10% O_2_, N_2_ at 350 °C for 40 min, and then cooled down to 50 °C rapidly with N_2_ purging. Subsequently, the catalysts were again heated from 50–600 °C with a rate of 10 °C/min in N_2_.

In situ DRIFTS investigations were performed using a Nicolet 6700 spectrometer at 4 cm^−1^ resolution with 64 co-added scans. Prior to adsorption experiments, the catalysts were pretreated at 500 °C for 0.5 h in N_2_ (100 mL·min^−1^) to eliminate the physisorbed water and other impurities. Then the samples were cooled down to 350 °C. After the background was subtracted, the samples were firstly exposed to certain reaction gas mixtures containing 400 ppm NO, 10% O_2_, 1% H_2_O, and balanced with N_2_ (total flow 100 mL·min^−1^) for 40 min. Subsequently, the catalysts were treated under 400 ppm NO, 10% O_2_, 1% H_2_O, 100 ppm SO_2_, and balanced with N_2_ for 60 min, and the in situ DRIFTS spectra were recorded in the range of 4000–900 cm^−1^.

## 4. Conclusions

In this work, catalytic oxidation of NO over MnO_x_–CeO_2_ and MnO_x_–TiO_2_ catalysts were studied in the absence or presence of SO_2_. The optimal molar ratio of Mn/Ce and Mn/Ti are 0.7 and 0.5, respectively. MnO_x_–CeO_2_ catalyst gives the highest NO oxidation efficiency of 72% at 325 °C and the NO oxidation efficiency maintained unchanged in 5 h in the presence of 100 ppm SO_2_ at 350 °C, while MnO_x_–TiO_2_ catalyst only yields 62% NO oxidation efficiency at 375 °C, and exhibits poor catalytic activity below 325 °C. MnO_x_–CeO_2_ catalysts exhibit better catalytic activity and resistance to SO_2_ poisoning than that of MnO_x_–TiO_2_ catalysts, which is attributed that MnO_x_–CeO_2_ catalyst possesses higher surface area, better dispersion of MnO_x_ and stronger NO_x_ adsorption oxidation ability, offers the abundance of Mn^3+^ and more surface adsorbed oxygen, and SO_2_ might be preferentially adsorbed to the surface of CeO_2_ to form sulfate species, lessening the sulfation of MnO_x_ sites.

## Figures and Tables

**Table 1:** BET surface area of the catalysts.

Catalysts	Surface Area (m^2^/g)
MnO_x_–CeO_2_-0.7	93.17
MnO_x_–CeO_2_-0.7-S	67.92
MnO_x_–TiO_2_-0.5	60.21
MnO_x_–TiO_2_-0.5-S	39.71

**Table 2:** Surface atomic distributions of the catalysts by XPS.

Catalysts	Atomic Concentration (%)			Surface Atomic Ratio (%)		
	Mn	Ce or Ti	O	Mn^3+^/(Mn^2+^ + Mn^3+^ + Mn^4+^)	Ce^3+^/(Ce^3+^ + Ce^4+^) or Ti^3+^/(Ti^3+^ + Ti^4+^)	O_α_/(O_α_ + O_β_)
MnO_x_–CeO_2_-0.7	5.6	26.4	68.0	46.42	41.46	33.4
MnO_x_–CeO_2_-0.7-S	5.4	19.7	70.6	-	25.2	50.3
MnO_x_–TiO_2_-0.5	13.6	18.8	67.6	47.31	87.9	25.6
MnO_x_–TiO_2_-0.5-S	9.4	13.3	74.4	-	87.1	55.8
